# A multi-level intervention in subsidized housing sites to increase fruit and vegetable access and intake: Rationale, design and methods of the ‘Live Well, Viva Bien’ cluster randomized trial

**DOI:** 10.1186/s12889-016-3141-7

**Published:** 2016-06-28

**Authors:** Kim M. Gans, Gemma Gorham, Patricia M. Risica, Akilah Dulin-Keita, Laura Dionne, Tina Gao, Sarah Peters, Ludovica Principato

**Affiliations:** Institute for Community Health Promotion, Brown University School of Public Health, Providence, RI 02912 USA; Department of Human Development and Family Studies and Center for Health Interventions and Prevention, University of Connecticut, Storrs, CT 06269 USA; Department of Management, Sapienza University of Rome, Sapienza University, Rome, Italy

**Keywords:** Diet, Food access, Fruit and vegetable, Farmer’s market, Mobile market, Nutrition education: Low income

## Abstract

**Background:**

Adequate fruit and vegetable (F&V) intake is important for disease prevention. Yet, most Americans, especially low-income and racial/ethnic minorities, do not eat adequate amounts. These disparities are partly attributable to food environments in low-income neighborhoods where residents often have limited access to affordable, healthful food and easy access to inexpensive, unhealthful foods. Increasing access to affordable healthful food in underserved neighborhoods through mobile markets is a promising, year-round strategy for improving dietary behaviors and reducing F&V intake disparities. However, to date, there have been no randomized controlled trials studying their effectiveness. The objective of the ‘Live Well, Viva Bien’ (LWVB) cluster randomized controlled trial is to evaluate the efficacy of a multicomponent mobile market intervention at increasing F&V intake among residents of subsidized housing complexes.

**Methods/Design:**

One housing complex served as a pilot site for the intervention group and the remaining 14 demographically-matched sites were randomized into either the intervention or control group. The intervention group received bimonthly, discount, mobile, fresh F&V markets in conjunction with a nutrition education intervention (two F&V campaigns, newsletters, DVDs and cooking demonstrations) for 12 months. The control group received physical activity and stress reduction interventions. Outcome measures include F&V intake (measured by two validated F&V screeners at baseline, six-month and twelve-months) along with potential psychosocial mediating variables. Extensive quantitative and qualitative process evaluation was also conducted throughout the study.

**Discussion:**

Modifying neighborhood food environments in ways that increase access to affordable, healthful food is a promising strategy for improving dietary behaviors among low-income, racial and ethnic minority groups at increased risk for obesity and other food-related chronic diseases. Discount, mobile F&V markets address all the major barriers to eating more F&V (high cost, poor quality, limited access and limited time to shop and cook) and provide a year-round solution to limited access to healthful food in low-income neighborhoods. LWVB is the first randomized controlled trial evaluating the effectiveness of mobile markets at increasing F&V intake. If proven efficacious at increasing F&V consumption, LWVB could be disseminated widely to neighborhoods that have low access to fresh F&V.

**Trials registration:**

Clinicatrials.gov registration number: NCT02669472 First Received: January 19, 2016.

**Electronic supplementary material:**

The online version of this article (doi:10.1186/s12889-016-3141-7) contains supplementary material, which is available to authorized users.

## Background

Eating ample amounts of fruits and vegetables (F&V) is associated with a lower risk for many chronic diseases [1–8] and may also help with weight management [2, 9–11]. The Dietary Guidelines for Americans recommend a daily intake of 3 ½ to 5 cups of F&V [12] Yet, most U.S. adults fall short of these recommendations, [13–15] with low income and some racial/ethnic minority groups even less likely to eat the recommended amount of F&V [16–25].

These disparities are partly attributable to the food environments in low-socioeconomic status (SES) and racial/ethnic minority neighborhoods, where residents often have limited access to affordable, healthful, high quality food and easy access to inexpensive, unhealthful food [19, 22, 23, 25–29]. Retail food outlets in these neighborhoods are generally less likely to stock healthful foods, more likely to offer lower quality produce, and more likely to have higher prices than stores in higher-income or predominantly Non-Hispanic white neighborhoods [30–41].

These findings support previous quantitative and qualitative studies, which identified the major barriers to eating more F&V as high cost, poor quality and limited access [20, 25, 42–46]. Other documented barriers to eating more F&V are hectic lifestyles (leaving little time for shopping and cooking), food preferences, negative attitudes and perceived norms regarding healthy eating, and lack of knowledge, skills, self-efficacy and social support [47–56]. Although individuals’ dietary choices are personal decisions, they are made within a complex mix of social and environmental influences that can make healthier choices more or less easy, accessible, and affordable [57–59]. Thus, altering environmental and social influences on eating behaviors is a promising strategy that may be more generalizable, cost-effective, and sustainable than individual or group behavior change interventions [34, 60–62].

Farmer’s markets and mobile F&V markets have emerged as an innovative approach for increasing access to healthful food [49, 63]. Although such markets demonstrate potential for improving dietary intake, there have been no randomized trials studying their efficacy to date. Additionally, while environmental interventions such as mobile markets may improve F&V intake by increasing access, a multi-level approach that also delivers behavioral interventions to increase motivation and skills and to decrease personal barriers to increasing F&V intake may be the most effective approach [64, 65]. The work of Dibsdall et al., found that for low SES adults, access and affordability were only two of the problems surrounding low F&V consumption and that personal determinants needed to be addressed as well [66]. Thus, research is needed on practical, cost-effective interventions that not only improve F&V access and affordability but also change personal determinants such as knowledge, skills, perceived barriers, attitudes, self efficacy and perceived social support [16, 59]. The purpose of this paper is to describe the intervention development, protocols and measures used in the ‘Live Well, Viva Bien’ (LWVB) research study.

LWVB is a cluster randomized controlled trial in 15 subsidized housing complexes designed to evaluate the efficacy of a multicomponent intervention that includes discount, mobile fresh F&V markets--*‘Fresh To You’(FTY)* —in conjunction with a nutrition education intervention. The primary aims of this study are to: 1) Conduct formative research with residents living in subsidized housing projects to inform the multi-level intervention and; 2) Implement a cluster randomized trial to study the efficacy of the FTY markets combined with the educational/motivational interventions at increasing F&V access, availability, and consumption compared to a Comparison intervention (attention placebo).

In addressing the aforementioned specific aims, we also have the opportunity to examine mechanisms involved in the delivery and receipt of programming and the potential causal effects of the intervention. To that end, we are addressing the following secondary aims: 1) To include extensive implementation process evaluation to determine costs, reach, fidelity and dose and the relationship of these variables with evaluation outcomes; and to 2) Use a mediating variable framework to examine relationships among important psychosocial factors/determinants with changes in F&V consumption.

## Methods

### Overview of study design

All study activities occur at housing sites in Providence County, Rhode Island. Pre-intervention focus groups were conducted with housing complex residents (from non-study sites) to inform intervention development. A total of 15 subsidized housing complexes were recruited into the evaluation cohort. One smaller housing complex served as a pilot site for the intervention group and the remaining fourteen demographically-matched sites were randomized into either the intervention or control group. Adult residents from each housing site were recruited for the evaluation cohort prior to randomization. The multicomponent intervention lasts one year and includes baseline, 6 and 12 month follow-up surveys as well as extensive quantitative and qualitative process evaluation throughout the course of the study. Participants are given a $30 gift card incentive upon completion of each of the three surveys ($90 total). All study protocols were approved by the Brown University Institutional Review Board and all participants provided verbal informed consent. A detailed description of the intervention and evaluation follows.

### Recruitment of housing sites

A recruitment brochure delineating the details of the study and the benefits for participating sites was distributed to subsidized housing complexes in Providence County, Rhode Island that had at least 190 units. Meetings were held with housing complex administrators and staff at potential sites to discuss the study and its benefits, as well as to ascertain their interest in participating. A housing authority official from each interested housing site signed a Memorandum of Agreement, which delineated the responsibilities of the researchers and the participating housing authority. A Resident Assistant from each housing site was hired to assist with recruitment and intervention activities.

### Eligibility criteria for housing sites

In order to participate in the study, the housing complex needed to have: at least 190 units; a relatively low turnover rate (<20 %); a community room or center where surveys, markets and other intervention activities could occur; a willingness to be randomized into one of the two experimental conditions, support the study activities for the duration of the study and help Brown research staff recruit and hire an on-site Resident Assistant. In addition, at least 90 % of the residents needed to be able to speak and read either English or Spanish.

Housing sites were recruited into the study in pairs, matched by number of units, type of site (family or elderly/disabled) and race/ethnicity. The first seven participating sites were Providence Housing Authority sites. The next four sites were Pawtucket Housing Authority Sites and the last four sites were Woonsocket Housing Authority Sites. Nine sites were elderly/disabled housing sites and six were family sites.

### Recruitment of study participants

Participant recruitment at each housing complex lasted for approximately one to three months and began with an on-site recruitment event in the housing complex community room. In advance of an event, Brown research staff and the Resident Assistant displayed posters and door hangers throughout the complex to advertise the recruitment event, study details, and a toll-free number to call with any questions.

On the day of the recruitment event, Brown research staff set up tables in the housing complex community room. An Excel sheet log that included a list of all room numbers in the housing complex was given to the Resident Assistant, who was seated at a table at the entrance to the community room. She greeted residents, verified that they lived in the housing complex and checked off their room number on the Excel sheet. Residents were then escorted to the tables where Brown’s bilingual research staff further screened them to ensure that they met all of the eligibility criteria. If found to be eligible and interested in participating, residents consented to participate and were enrolled in the study.

### Eligibility criteria of participants

To participate in the evaluation study, participants needed to: be 18 years of age or older; live in the housing complex as a full-time resident; shop for their household’s food at least half of the time, not have any major medical conditions that would prevent them from participating in study activities or events; not be planning to move in the next year; be able to read and understand either English or Spanish; and have access to a Digital Video Disk (DVD) player (or computer that could play DVDs). If housing residents were not eligible or did not choose to be in the evaluation study, they were still invited to participate in the intervention activities.

### Baseline surveys

Baseline surveys were either conducted by Brown research staff, either in-person at the housing complex, or by phone via computer-assisted telephone interviewing. All baseline data that was collected in person was first reviewed for completeness by Brown data clerks and then entered into the online computer database.

### Randomization

**Fig. 1:**
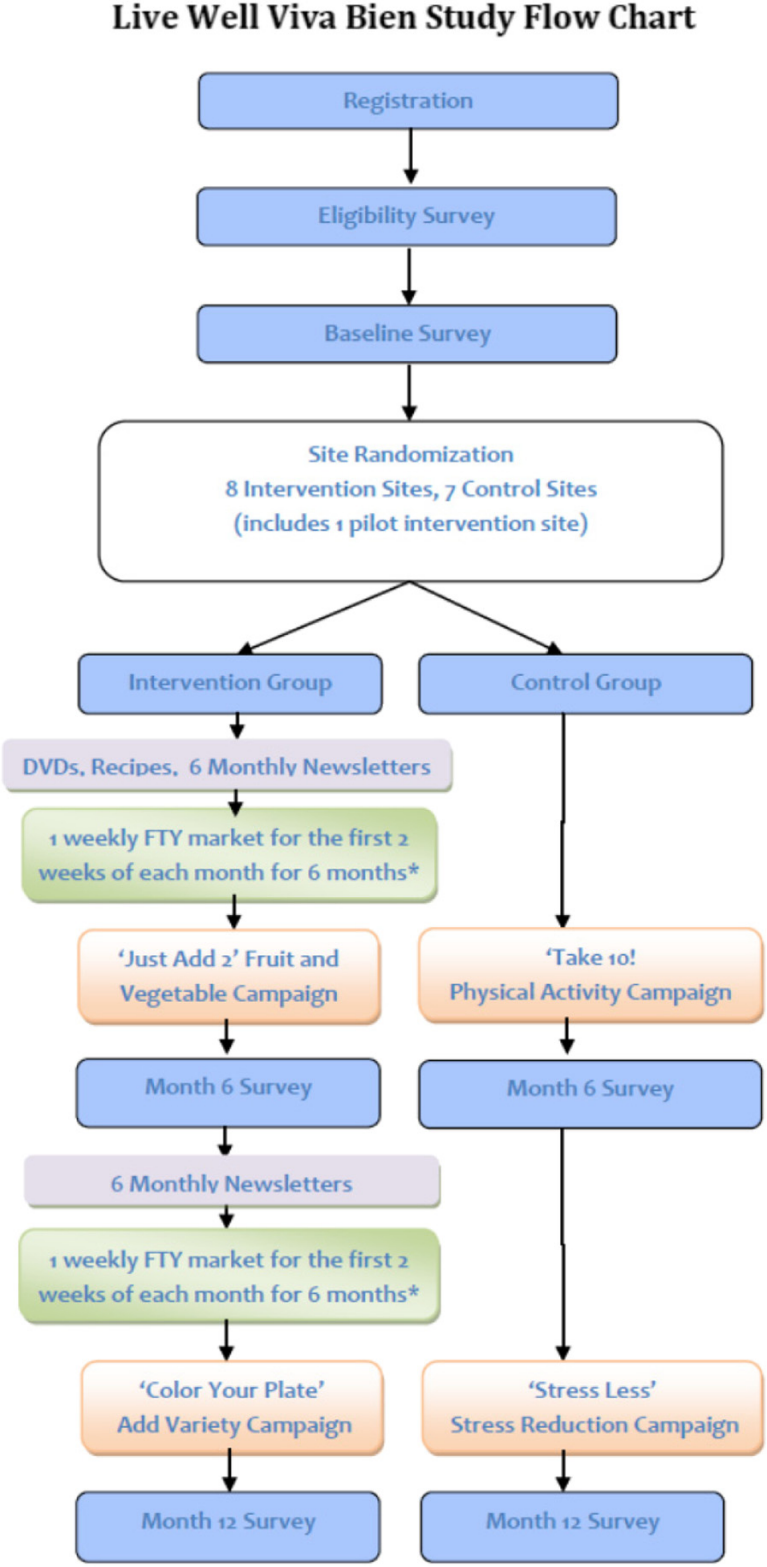
Live Well Viva Bien study flow chart

After baseline surveys were completed at each matched pair of sites, the Brown Data Manager assigned the sites to either the intervention or control group using a random number generating function in Excel. *See Fig.**1**for the Live Well Viva Bien study flow diagram.*

## Intervention

### Formative research and intervention development

We conducted 10 focus groups (eight exploratory and two confirmatory) at three housing sites with low-income, racially and ethnically diverse residents of subsidized housing complexes (sites not included in the main trial) to inform the development of the intervention. The focus groups lasted one to two hours, were recorded and were led by trained facilitators using standard focus group procedures [67]. Refreshments were served and participants received a $25 gift card.

The first eight exploratory focus groups (four in English and four in Spanish) asked participants about personal and environmental barriers and facilitators associated with purchasing, preparing and consuming more F&V, where they usually shopped and how they felt about the cost, quality and accessibility of F&V in their neighborhoods. The facilitators then explained the proposed intervention and gathered participants’ input and suggestions about each component of the intervention including where and when the markets should be held at the housing complex, the types of produce they would like to see sold at the markets, the best ways to publicize the markets and campaigns, the nutrition education topics that would be most helpful to them, the length and content of the DVDs, types of incentives and/or prizes to offer at the campaigns, and etcetera.

At the end of each focus group, the recordings were transcribed, translated and summarized. The findings were used to inform the development of the recruitment, marketing and intervention materials. After the first draft of the materials were developed, two confirmatory focus groups were held (one in English and one in Spanish) during which participants provided more input into both the content and design of the intervention materials.

A total of 79 participants participated in the formative research: 72 % were female, 39 % were Hispanic/Latino (47 % identified as Dominican). A total of 35 % were White, 27 % were Black/African American, 14 % were mixed race, 48 % were unemployed, 73 % had a high school education or less, and mean age was 59 years.

From these groups we learned that the high cost of F&V, limited access to high quality, affordable F&V in their neighborhoods and limited time to shop and cook were the major barriers to eating more F&V. Thus, specific educational content to address these barriers were included in the intervention materials. Focus group participants expressed interest in learning more about how to prepare F&V, so we created and distributed recipe cards as well as DVD segments on how to prepare F&V and the DVDs used a cooking show format as focus group participants suggested. They also expressed interest in knowing what an appropriate serving size was for F&V as well as the health benefits of eating more F&V; therefore, this information was also included in the intervention materials. When asked about the type of incentives that would most motivate them to participate in the study, they told us Walmart or supermarket gift cards, which is what we chose to use.

Focus group participants gave us the following suggestions for encouraging participation in the FTY markets: ensuring that the produce at the markets was sold at no more than retail price, preferably lower; that it was high quality/fresh; that the markets accepted debit and Electronic Benefits Transfer (EBT) cards; and that residents knew when and where the markets would be held through the use of posters and flyers at frequented locations in the housing complexes. They also gave us a list of the types of F&V they would like to purchase. All of these suggestions were incorporated into the markets.

At the confirmatory focus groups, where we shared drafts of the educational intervention materials and DVDs, participants suggested focusing on one topic per newsletter and using photos instead of cartoon images. For the DVDs, in addition to including cooking show segments, they also suggested sharing the experiences of real people with follow-ups on their progress in subsequent DVDs. They also wanted the DVDs to include multiple short segments rather than a long segment, so they could easily skip from one segment to another. We implemented all of these suggestions in the development of the intervention materials.

### Intervention framework

This multicomponent intervention is based on a social ecological model, which offers a conceptual framework that recognizes that behavior is affected by multiple levels of influence and that an intervention will be most effective when it targets changes in multiple levels or domains [68–70]. The multi-level LWVB intervention operates within the intrapersonal/individual, interpersonal (social), and environmental domains.

The FTY markets focus on changing the physical food environment by increasing access and availability to fresh F&V. The motivational/educational components of the FTY intervention focus on individual and interpersonal factors by enhancing opportunities for social support and networking, increasing opportunities for participants to role model eating F&V and changing perceived norms to support increased consumption of F&V. The intervention also aims to change the informational environment of the housing site by providing regular communication channels through which educational information about healthy eating is distributed, thereby increasing the awareness of the importance and the benefits of eating F&V. The educational materials also aim to change participants’ personal determinants including increasing knowledge, attitudes, skills, self-efficacy, readiness to change, positive outcome expectancies/perceived benefits, taste perceptions, and perceived social support, while decreasing perceived barriers to eating more F&V.

**Fig. 2:**
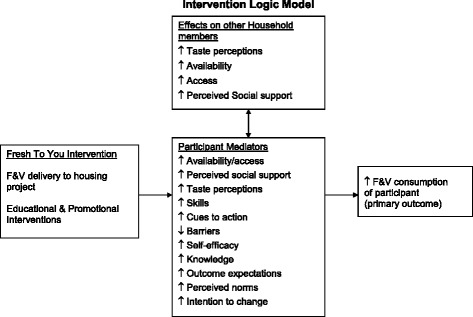
Intervention logic model

The theoretical framework for the LWVB intervention is the Social Cognitive Theory (SCT), which defines behavior as a triadic, dynamic, and reciprocal interaction of personal factors, behavior and the environment [71–73]. Based on SCT, behavior change is facilitated if the individual believes change is possible, has an opportunity to develop and practice new skills, and receives support from the environment. Reciprocal determinism explains the interaction between personal factors, behavior and the environment. Behavior changes induced by changes in the individual and in the environment are more likely to be effective and sustained. Thus, the FTY intervention specifically targets changes in personal determinants and interpersonal determinants as well as the availability of and access to F&V in the environment. Both the DVDs and the newsletters include testimonials from target audience members discussing benefits that they gained from eating more F&V to improve outcome expectatations/perceived benefits. The newsletters and DVDs provide ideas on how to overcome barriers such as cost, lack of time, lack of social support, lack of cooking skills, etcetera . Role modeling of success stories in newsletters and testimonials in DVDs aims to increase self-efficacy. Moreover, the campaigns incorporate goal-setting, feedback and self regulation techniques such as self-monitoring and problem-solving around barriers. This intervention addresses external/environmental determinants by increasing F&V access and availability directly with the markets. In addition, participants see neighbors purchasing F&V at the markets, which could change perceived norms and social support. The intervention also attempts to change taste perceptions of F&V through the taste-testing demonstrations, recipes and the provision of fresh, high quality F&V. See the Logic Model in Fig. 2.

### Intervention components

#### ‘Fresh To You’(FTY) discount, fresh fruit and vegetable markets

The intervention sites receive regularly-scheduled, discount, fresh F&V markets for one year. These FTY markets are not farmer’s markets, but rather mobile markets selling both local and non-local produce on a year-round basis at prices at or below local supermarket prices [74]. For the current study, the original plan to bring the markets to each housing complex on a weekly basis changed as we learned through the pilot that most residents only shopped at the markets the first two weeks of the month, consistent with disbursement of Supplemental Nutrition Asssistance Program (SNAP) benefits. Therefore, at the next seven (7) intervention sites, markets were only scheduled during the first two weeks of each month.

Prior to starting the markets, Brown research staff met with the designated site contacts, who helped determine the best times and locations for the markets. On a mutually agreed upon schedule, our produce distributor brings FTY markets carrying between 50 to 70 different produce items, including staples (e.g., potatoes, onions, carrots, celery, tomatoes and bananas); seasonal items (e.g.,clementines in the winter and peaches and blueberries in the summer); culturally desired ethnic produce items (e.g., plaintains, yucca and mangoes) and exotic produce (e.g. Asian pears and purple eggplant) to introduce new F&V to residents.

The majority of the FTY markets at the senior and disabled housing complexes are held indoors as it is difficult for many of these residents to get outside to shop. Markets are also held indoors at family housing complexes during inclement weather. F&V are arranged in bins on the tables in a central location. A check-out station is set up on a separate table that includes a scale for produce weighing and a Point-of-Sale (POS) system that serves as a cash register and a data collection and reporting unit.

Our produce distributor retrofitted a car trailer to serve as an outdoor, ‘mobile fresh F&V market’ (See Additional file 1: Market Photos) and, in good weather, the markets are held outside at the family housing complexes*.* This retrofitted trailer is pulled by a van and brought to each of the intervention group housing sites at pre-determined days and times during the first two weeks of each month on a regularly scheduled basis. The produce items are set up on racks affixed to three sides of the trailer and shoppers enter through the side and rear doors and exit through the rear door. The scale and POS system are set up on a table outside of the rear door of the trailer and shoppers pay for their produce here. FTY markets accept cash, debit/credit cards and EBT cards (SNAP benefits). Each market lasts two hours and the produce is sold at or below retail price. On average, FTY prices are 15 % to 25 % lower than local retail supermarket prices.

Brown University research staff, with the help of a graphic designer, created a logo for the FTY markets, which is used on all promotional materials. Signs, posters, doorhangers and flyers are used to advertise the markets. In addition, the Resident Assistant at each site and Brown research staff often circulate throughout the housing complex on market days to encourage residents to come to the markets.

The FTY intervention at each site begins with a highly-publicized ‘Kick-Off’ event, which includes the first FTY market, chef-run cooking demonstrations and taste-testing events, along with recipes and detailed information about the upcoming intervention activities. Brown research staff also recruit residents into the first, six-week campaign at the Kick-Off events.

#### Motivational/Educational intervention

Intervention materials are delivered to residents of each housing complex before the first market is held. Each resident household in the intervention housing complex receives a large, reusable shopping bag with the ‘Fresh To You’ logo on it that contains a binder with the following materials: a ‘Welcome Page’, which provides an overview of the intervention, the first month’s newsletter, three DVDs, 48 recipe cards and enough three-hole binder sleeves to store the remaining newsletters they will receive. All materials are provided in English and Spanish. A description of each of the motivational/educational intervention components follows.

#### Campaigns

The intervention includes two educational/motivational campaigns: the first campaign, *‘Just Add 2’*, begins at the Kick-Off event at each site and the second campaign, entitled *‘Color Your Plate’*, is held approximately one month after the six-month follow-up surveys are completed at each site. Both campaigns include full-color campaign booklets with goal-setting activities, educational and motivational content, and F&V trackers that ask participants to record the number of F&V they eat each week. These booklets are worded at a 5^th^ grade reading level. Participants are instructed to read one section of the booklet and to complete the weekly activities included in that section. At the end of the week, participants are asked to deposit their weekly trackers into a raffle box kept at each site. A midpoint event and a final event are also held as part of the campaigns with chef-led cooking demonstrations and taste-testing events (see below). At these events, Brown research staff pull several participants’ trackers out of the raffle box and distribute prizes (blenders, microwaves, woks, crock pots, etc.) to the winners. Approximately $500 in incentive prizes are given away at each site. The campaigns are designed to be ‘self-directed’; however, Brown research staff attend the FTY markets to answer any questions.

*‘Just Add 2’,* the first, six-week kick-off campaign, was designed to increase participants’ F&V consumption by two servings by the end. Participants receive a full-color, campaign booklet, which includes: a description of the campaign; instructions; a table of recommended daily F&V intake (in cups) by age, gender and activity level; photos depicting what a one-cup serving looks like for different F&V; a goal-setting form; the weekly F&V tracker as well as motivational and informational, tips and interactive weekly activities. The weekly chapters and activities are as follows: 1: Overcoming Common Roadblocks; 2: Fruits &Vegetables with Meals and Snacks; 3: Fruits &Vegetables on the Go; 4: Shopping for Fruits &Vegetables; 6: Try Something New and 7: Staying on Track.

*‘Color Your Plate’,* the second, six-week campaign, is focused on increasing the variety of F&V that participants eat. Campaign participants receive a colorful campaign booklet that includes a F&V tracker as well as educational and motivational content for each of the six weeks. Each week focuses on a specific color F&V; the last week focused on variety. The weekly chapters are: 1. Red; 2. Yellow and Orange; 3. Green; 4. Blue and Purple; 5. White and Brown; and 6. Overall Variety. Each week’s section includes a table listing the F&V of the color for that week, along with information on how to choose and store each F&V and when they are in season. Columns are also included on the table for participants to check and indicate whether they like or dislike each F&V and whether they have ever tried it. Each weekly section also includes a daily chart where participants can record how many F&V of that color they eat each day of the week. Then, at the end of the week, participants are asked to add up the numbers in this chart and write the total number on the weekly F&V tracker, which they deposit into the raffle box for prize drawings. Each section also includes tips for how to eat more of that color F&V during the week and the associated health benefits of those F&V.

#### Videos/DVDs

Three, 20-min DVDs were created to support and encourage residents in the intervention group to increase their F&V intake. Each DVD includes a menu so participants can easily navigate to specific segments. The first DVD includes information about the FTY markets; cooking demonstrations showing how to prepare healthy meals for less than $6.00; success stories about real people and health benefits associated with eating more F&V. The second DVD includes guidance and a cooking demonstration regarding how to plan quick, healthy meals; additional success stories about real people; information regarding how to save money while eating healthy; how to involve family members in healthy eating; how to find time to eat healthy, as well as a feature on preparing jicama, yucca, leeks, and mangoes. The third DVD includes another cooking demonstration; more examples of how to prepare quick, inexpensive, healthy meals; additional success stories about real people; and a feature on preparing spaghetti squash and starfruit.

#### Monthly newsletters

A two-page, full-color monthly newsletter is delivered to the door of each intervention housing complex resident on the first day of each month either by the onsite Resident Assistant or Brown research staff. Each newsletter highlights a particular F&V in season that month as well as key nutrients and health benefits associated with the featured F&V and information on how to choose and store them. The newsletters also include a section that lists other F&V in season that month and a section explaining how to save time and money when buying and preparing these F&V. The back side of each newsletter includes a recipe for the F&V of the month with colorful photos of the completed recipe.

#### Recipe cards

A total of 48 recipe cards were created that correlate with the cooking demonstrations on the DVDs (described above). These recipes were chosen because they were easy-to-follow, healthy, relatively quick to prepare, inexpensive and included culturally desired ethnic foods such as jicama, yucca, plantains and mangoes. All recipes were provided in both English and Spanish.

#### Cooking demonstrations

A trained chef visited the housing sites six times during the one year intervention period before, during and after the campaigns on the day of a market. She presented half-hour long cooking demonstrations that featured at least one fruit or vegetable, explaining to participants how to prepare it. One of our bilingual staff translated for the Spanish-speaking participants. All participants received a copy of the recipe that was demonstrated. All of the F&V in the recipes were available at that day’s market.

### Comparison/control intervention

Brown University contracted with the Greater Providence YMCA to provide a physical activity and stress reduction intervention at the 7 housing complexes in the comparison group. Two, six-week campaigns were developed jointly by the Brown study team and YMCA staff. These campaigns follow the same format as the intervention group campaigns and are provided during the same time periods as those at the intervention sites. Campaign participants also receive a free, 6-week membership to the YMCA.

#### ‘Take 10!’ Campaign

The first comparison group campaign is ‘*Take 10!*’ which aims to increase participants’ daily physical activity by 10 min per day until participants reach the goal of at least 30–60 min per day. Participants are given a campaign booklet that includes six weekly sections focused on a particular goal and included: educational and motivational content, a goal setting and action step form, tips and suggestions for how to achieve that goal and the benefits associated with reaching the goal. The focus areas for each week are: 1. Move More, Sit Less; 2. Strength Training; 3. Increase Flexibility; 4. Walk More; 5. Add Intensity; and 6. Make Simple Changes.

Each week, participants are encouraged to read through that week’s section of the booklet, review their weekly goals and do the activities included in that week’s ‘Action Plan’ along with the bonus point activity. At the end of each day, participants are asked to write down the total number of minutes they spent being physically active on the Activity Tracking Sheet. Then, at the end of each week, they are asked to fill out the Weekly Raffle Form and return it in order to earn points toward the incentive prizes. For every ten points earned, participants receive a raffle ticket that is entered into a drawing at the midpoint and final events. Approximately $500 in incentive prizes was given away at each site.

#### ‘Stress Less’ Campaign

The second comparison group campaign was ‘*Stress Less’*, which aims to help participants reduce the amount of stress that they experience by adding stress reduction activities into their daily routines. Campaign participants are given a booklet that includes six weekly sections focused on a particular stress reduction technique. Each section includes: educational and motivational content, a goal setting and action step form, tips and suggestions for how to achieve that goal and the benefits associated with reaching the goal. The focus areas for each week are: 1. Muscle Relaxation and Tension Release; 2. Visualization; 3. Adequate Sleep; 4. Mindfulness and Meditation; 5. Time Management for Stress Reduction; and 6. Music for Relaxation. Each time the participants tries one of the recommendations, they record it on their Activity Tracking Sheet along with how they feel about the techniques. Then, at the end of the week, they are asked to turn in a completed Weekly Raffle form to receive points for each activity they tried and receive raffle tickets for incentive prizes. Raffle tickets are drawn at the midpoint and final events with approximately $500 in incentive prizes given away at each site.

## Process evaluation

Research staff collect and compile attendance counts for each of the campaign events at each site. Detailed FTY market sales data, including total sales, number of shoppers, items purchased and tender types are captured by the FTY POS cashiering system. Research staff record the number of participants who participate in the campaigns and other activities. The 6- and 12-month follow-up surveys include questions about participation in each component of the intervention including frequency of shopping at the FTY markets; perceptions of prices, quality and availability of F&V; participation in, or reasons for not participating in, each campaign; helpfulness of each campaign; participation in, and usefulness of, the cooking demonstration and taste testing events; and the use and usefulness of the recipes, newsletters, and DVDs. Participants are also asked about participant and family changes in shopping behaviors and F&V intake behaviors as a result of the markets. The follow-up surveys also include open-ended questions that ask participants about what they learned and the behavioral changes that they made as a result of the intervention as well as suggestions for how the intervention could be improved.

## Evaluation measures

### Primary outcome measure

The primary outcome is F&V intake, which is measured using two validated instruments: a Two-Item Cup F&V intake screener, [75] and the 18 item National Cancer Institute (NCI) Eating at America’s Table All Day Screener [76]. The two-item measure consists of two questions: “About how many cups of fruit (including 100 % pure fruit juice) do you eat or drink each day?” and, “About how many cups of vegetables (including 100 % vegetable juice) do you eat or drink each day?” Each question includes a description of the amount of F&V in one cup to aid participants in choosing their portion size. Portion size options range from none to 4 cups or more.

The NCI All Day Screener queries foods consumed over the past month. Participants are asked to think about the F&V they usually ate last month and to report the frequency (from never to 5 or more times per day) and serving size (from less than ½ cup to more than 1 ½ cups) for each F&V. Questions are asked about 100 % fruit juice, fruit, salad, french fries or fried potatoes, other white potatoes, cooked beans, other vegetables, tomato sauce, vegetable soup and mixtures that include vegetables. To calculate F&V intake, the response options are standarized to a daily cup serving by multiplying the frequency of consumption by the portion size. The total consumption of F&V is calculated by summing the products of each food group. The responses to the frequency questions are recoded to daily averages based on standard NCI methods [76].

In addition to these two measures, F&V practices are assessed in a series of eleven F&V habits questions adapted from previous questionnaires [51, 77, 78]. These questions include how often in the past few months they: ate fruit at breakfast; added vegetables to breakfast dishes; ate more than one type of fruit per day; ate more than one type of vegetable per day; ate a lettuce-based salad or vegetable at lunch; ate fruit at lunch; eat a lettuce-based salad or vegetable at dinner; ate two or more different vegetables or a vegetable and a salad at dinner; added vegetables to other foods or dishes; ate fruit or vegetables as a snack in-between meals; and ate just fruit as dessert instead of a rich dessert. All questions have five levels of response (always, often, sometimes, rarely or never). The sum of all responses is taken to get the total Fruit and Vegetable Habits Questionnaire score. All items are scored so that higher scores are indicative of higher F&V intake behaviors.

### Demographic measures

Demographic measures include gender, age, marital status, country of birth, years lived in the United States, language(s) spoken in the home, ethnicity (Hispanic or not), race, employment status, education, household composition, income, and participation in food assistance programs.

### Potential mediating variables

Knowledge of Fruit and Vegetable Intake: Two questions adapted from previously published questionaires [79–81] are included in the baseline and follow-up surveys to assess knowledge of the appropriate amount of fruits and vegetables an adult should eat. We modified the response categories from servings to cups to be consistent with national dietary guidelines and the NCI screener. The questions are: “How many cups of fruit do you think someone your age should eat each day to have a healthy diet?” and “How many cups of vegetables do you think someone your age should eat each day to have a healthy diet?”

Fruit and Vegetable Availability: Twenty (20) questions regarding F&V availability are included in the baseline and follow-up surveys. These questions were modified from the “Home Environment Survey” [82] to include additional culturally-desired ethnic F&V such as plantains, mangoes, yucca, etc. The questions read as follows: “How often are (type of F&V) available in your home?” Responses range on a five-point Likert scale from never to always.

Barriers to Eating More Fruits and Vegetables: Seven questions to assess barriers to eating more F&V are included in the baseline and follow-up surveys in the form of a statement. Participants are asked to choose how much they agree or disagree with each statement. Responses range on a five-point Likert scale from agree a lot to disagree a lot. The specific barriers queried were drawn from previous studies [55, 83–86] and from our own formative research and include: lack of knowledge regarding how to prepare F&V; lack of time to shop and prepare F&V; high cost of F&V; lack of access to stores selling F&V; F&V spoil too quickly and family preferences.

Perceived Benefits/Outcome Expectations: Two questions regarding perceived benefits/outcome expectations associated with eating more F&V are included in the baseline and follow-up surveys with responses ranging on a five-point Likert scale from agree a lot to disagree a lot. The specific questions are: “You are helping your body by eating more F&V.” and “You may develop health problems if you don’t eat enough F&V.”

Self-Efficacy for Fruit and Vegetable Intake: Six questions are drawn from the Townsend F&V Inventory [87, 88] to assess self-efficacy regarding F&V intake and included in the baseline and follow-up survyes. Responses are on a five point Likert scale and range from agree a lot to disagree a lot. The questions are: “Do you feel you can: eat more F&V as snacks; buy more F&V the next time you shop; plan meals or snacks with F&V; eat 2 or more servings of F&V with dinner; plan meals with more vegetables; and add extra vegetables to casseroles or stews.”

Stage of Change for Fruit and Vegetable Intake: Two questions to assess readiness to eat and readiness to buy more F&V were adapted from previous instruments [83, 89–92] and included in the baseline and follow-up surveys. Participants are asked to select the statement that best described them. Responses included: ‘You are not thinking about”, “You are thinking about”, You are definitely planning to”, “You are trying to” and “You are already eating or buying more F&V”. The responses correspond to these stages of change: pre-contemplation, contemplation, preparation, action and maintenance.

Social Support for Fruit and Vegetable Intake: Four questions regarding social support for F&V intake were adapted from previous research, [93] and included in the baseline and follow-up surveys. The first three questions are: “During the past three months, how often did your friends, family or household members encourage you to: 1) buy F&V; 2) eat F&V; and 3) serve your family more F&Vs. The last question is “During the past three months, how often did your friends, family or household members buy F&V for your household?”. Responses ranged on a five-point Likert scale from never to very often.

Importance of Buying/Eating more Fruits and Vegetables: Two questions regarding the importance of buying and eating more F&V were adapted from previous research [93] and included in the baseline and follow-up surveys. We adapted these questions to ask, “How important is it to you to buy more F&V?” and “How important is it to you to eat more F&V?” Responses range on a five-point Likert scale from not at all important to extremely important.

### Data analysis

To evaluate potential differences between groups that may have occured by chance in the random assignment process, demographics will be assessed by group using chi squared tests for categorical data and analysis of variance for continuous data. Also, group differences in baseline values for outcomes and mediators will be assessed using ANOVA models. To assess characteristics of the baseline F&V intake and mediators, outcomes and mediator variables will be assessed by demographic categories using ANOVA models. To examine F&V intake, mixed-model analysis of variance using SAS PROC MIXED will be used to account for a potential cluster effect within each housing site. All analyses will be performed using SAS version 9.4 (SAS Institute, Cary, NC).

### Sample size considerations

The original sample size projections showed that ending the study with 75 participants in each of 16 housing complex sites (8 sites per condition) using reasonable estimates of the intraclass correlation (0.01) and Standard Deviation 2.5 (based on our previous studies), we would be able to detect an effect size of 0.50 cups (based on F&V Screener change scores) between experimental groups with 80 % power. We originally planned to recruit 16 sites but dropped our sample size slightly (7 sites in each condition and 1 pilot site = 15 sites) because of large budget cuts from NCI at the beginning of the funding period. We anticipated retention at 75 % or greater of the original sample so aimed to enroll an average of at least 107 participants at each of the 15 housing sites.

## Discussion

‘Live Well, Viva Bien’ (LWVB) is the first cluster-randomized trial to study the efficacy of bringing mobile markets to low-income neighborhoods. Non-experimental studies have shown that seasonal farmers’ markets selling local produce can increase F&V intake of participants’ but these studies used cross-sectional or one-group, repeated-measures designs [16, 94–97]. Several other studies have examined the efficacy of educational programs and/or monetary vouchers for F&V at farmer’s markets; but did not study the efficacy of the markets themselves [98, 99].

Moreover, while year-round mobile produce markets have expanded nationwide, [49, 63, 100] and statistical models demonstrate their potential for increasing access to F&V, [101, 102] very few studies have done any evaluation of mobile markets and no rigorous evaluation has been conducted to demonstrate the efficacy of these markets in changing dietary behavior until now. One non-experimental study did demonstrate the effectiveness of ‘Veggie Mobile’, a van carrying fresh, discount-priced produce to low-income neighborhoods in New York State's Capital Region at increasing F&V intake among 43 low-income seniors in two senior housing complexes [95]. Another two-week long, non-experimental study found that the presence of a mobile fruit vendor increased children's F&V intake and reduced their consumption of unhealthy snacks [103]. Longitudinal research with larger sample sizes and more rigorous research designs including cluster randomized trials, such as LWVB, are needed to verify the direct and indirect impacts of mobile markets on F&V consumption behavior [16].

If the LWVB intervention is found to be effiacious in increasing F&V intake in comparison to the control group, it will be the first randomized trial to demonstrate the efficacy of farmer’s markets or mobile F&V markets in changing dietary behavior. These data are crucial to provide evidence to potential funders for fresh F&V market programs. One of the most important new potential sources of funding for food access and nutrition programs may be the community benefit obligations of U.S. nonprofit hospitals that seek federal tax exempt status [104, 105]. Federal community benefit standards have been in place since 1969; however, they underwent a significant transformation with the passage of the Affordable Care Act (ACA) in 2010 [106]. Nonprofit hospitals are now required to conduct a community health needs assessment (CHNA) every three years with community input, identify priority health needs, based on the results of the CHNA, develop a plan to address those needs, and submit an annual report of their community benefit activities that must include an evaluation [106]. In addition to establishing a framework for how to assess community needs, the new rules acknowledge that community benefits are more than simply a provision of financial assistance to low-income patients and improved access to care. The final rule, published in December 2014, states that hospitals may also consider “…the need to prevent illness, to ensure adequate nutrition, or to address social, behavioral, and environmental factors that influence health in the community” [106]. The specific incorporation of “nutrition” and “environmental factors” signals support for the idea that improving the community food environment and addressing barriers to healthy food access are viable strategies to improve population health that could be considered for funding by non-profit hospitals [107]. However, in order for interventions to be considered under this mechanism, they must be evidence-based. Thus, if the LWVB mobile market program is determined to be efficacious, it would provide evidence in support of the thousands of mobile markets around the country to be eligible for community benefit funds from their local hospitals.

Mobile markets like those in the LWVB study have the opportunity to not only increase F&V consumption, but also to increase food security because the prices of F&V are lower than supermarket prices. This benefit could be furthered with healthy food financing initatives, which provide grants to provide financial incentives for F&V to SNAP recipients, such as ‘Double Up Food Bucks’ (buy $15 of F&V and get a matching $15 coupon) [24, 108].

The results of LWVB will also inform the field about the importance of educational programming to coincide with F&V market programs. While the research design will not allow us to completely differentiate the effects of the markets vs. the educational programming, the process evaluation will allow for a dose–response analysis that could shed light on the relationship between participation in the markets and the various educational interventions on F&V intake. Additionally, this research may provide insight into the relative contributions of different components of a multi-level intervention toward increasing F&V consumption.

It is our belief that the findings from this study will make a substantial contribution to the field by providing important scientific evidence regarding the efficacy of mobile produce market programs. If there is ever to be a true and lasting impact on the unacceptably high prevalence rates of food-related chronic diseases, and their associated health disparities, we must identify evidence-based and place-based solutions that are well-accepted by the communities they serve and scientifically proven to be effective at changing health behaviors. This study will hopefully be able to identify and bring such an intervention to the forefront so that it can be widely disseminated to reach many more low-income, underserved neighborhoods and residents.

## Abbreviations

CHNA, Community Health Needs Assessment; DVD, Digital video disk; EBT, Electronic benefits transfer; F&V*,* Fruits and vegetables; FTY, Fresh to you; LWVB, Live Well, Viva Bien; NCI, National Cancer Institute; POS, Point-of-sale; SCT, Social cognitive theory; SES, Socioeconomic status; SNAP, Supplemental nutrition asssistance program

## Additional file

Additional file 1: Photos of the mobile market. (DOCX 7853 kb)

## References

[1] Hung H-C, Joshipura KJ, Jiang R, Hu FB, Hunter D, Smith-Warner SA, et al. Fruit and vegetable intake and risk of major chronic disease. J Natl Cancer Inst. 2004;96:1577–84. 10.1093/jnci/djh296 15523086

[2] Boeing H, Bechthold A, Bub A, Ellinger S, Haller D, Kroke A, et al. Critical review: vegetables and fruit in the prevention of chronic diseases. Eur J Nutr. 2012;51:637–63. 10.1007/s00394-012-0380-y PMC3419346 22684631

[3] Dauchet L, Amouyel P, Hercberg S, Dallongeville J (2006). Fruit and vegetable consumption and risk of coronary heart disease: a meta-analysis of cohort studies. J Nutr.

[4] Hartley L, Igbinedion E, Holmes J, Flowers N, Thorogood M, Clarke A, et al. Increased consumption of fruit and vegetables for the primary prevention of cardiovascular diseases. Cochrane Database Syst Rev. 2013;6:CD009874. 10.1002/14651858.CD009874.pub2 PMC6464871 23736950

[5] Wiseman M (2008). The second World Cancer Research Fund/American Institute for Cancer Research expert report. Food, nutrition, physical activity, and the prevention of cancer: a global perspective. Proc Nutr Soc.

[6] Fung TT, Stampfer MJ, Manson JE, Rexrode KM, Willett WC, Hu FB (2004). Prospective study of major dietary patterns and stroke risk in women. Stroke.

[7] Sorensen G, Linnan L, Hunt MK (2004). Worksite-based research and initiatives to increase fruit and vegetable consumption. Prev Med (Baltim).

[8] Hu FB, Manson JE, Stampfer MJ, Colditz G, Liu S, Solomon CG, et al. Diet, lifestyle, and the risk of type 2 diabetes mellitus in women. N Engl J Med. 2001;345:790–7. 10.1056/NEJMoa010492 11556298

[9] Alinia S, Hels O, Tetens I (2009). The potential association between fruit intake and body weight--a review. Obes Rev.

[10] Ledoux TA, Hingle MD, Baranowski T (2011). Relationship of fruit and vegetable intake with adiposity: a systematic review. Obes Rev.

[11] National Center for Chronic Disease Prevention and Health Promotion Division of Nutrition and Physical Activity, Centers for Disease Control. Research to Practice Series, No. 1 Can eating fruits and vegetables help people to manage their weight. (1):1–6. [National Center for Chronic Disease Prevention and Health Promotion Division of Nutriti http://www.cdc.gov/nccdphp/dnpa/nutrition/pdf/rtp_practitioner_10_07.pdf]. Accessed 21 Feb 2016.

[12] U.S. Department of Agriculture and U.S. Department of Health and Human Services. Dietary Guidelines for Americans, 2010. 7th Edition, Washington, DC: U.S. Government Printing Office; 2010. 10.3945/an.111.000430 PMC3090168 22332062

[13] McGuire S (2013). State Indicator Report on Fruits and Vegetables, 2013, Centers for Disease Control and Prevention, Atlanta, GA. Adv Nutr.

[14] Healthy People 2020 [https://www.healthypeople.gov/]. Accessed 21 Feb 2016.

[15] Adults Meeting Fruit and Vegetable Intake Recommendations — United States, 2013 [http://www.cdc.gov/mmwr/preview/mmwrhtml/mm6426a1.htm]. Accessed 21 Feb 2016. PMC4584842 26158351

[16] McCormack LA, Laska MN, Larson NI, Story M (2010). Review of the nutritional implications of farmers’ markets and community gardens: a call for evaluation and research efforts. J Am Diet Assoc.

[17] Guthrie J, Andrews M, Frazao E, Leibtag E, Lin B-H, Mancino L, Nord M, Prell M, Smallwood D, Variyam J, Ploeg M Ver: Can Food Stamps Do More To Improve Food Choices? An Economic Perspective. Economic Information Bulletin No. (EIB-29). 2007;2.

[18] Guenther PM, Juan W, Lino M, Hiza HA, Fungwe TV, Lucas R (2009). Diet Quality of Low-income and Higher-income Americans in 2003–2004 as Measured by the Healthy Eating Index-2005. FASEB J.

[19] Zenk SN, Schulz AJ, Hollis-Neely T, Campbell RT, Holmes N, Watkins G, et al. Fruit and vegetable intake in African Americans income and store characteristics. Am J Prev Med. 2005;29:1–9. 10.1016/j.amepre.2005.03.002 15958245

[20] Kamphuis CBM, Giskes K, de Bruijn G-J, Wendel-Vos W, Brug J, van Lenthe FJ (2006). Environmental determinants of fruit and vegetable consumption among adults: a systematic review. Br J Nutr.

[21] Bowman SA (2006). A comparison of the socioeconomic characteristics, dietary practices, and health status of women food shoppers with different food price attitudes. Nutr Res.

[22] Crump SR, Taylor BD, Sung JFC, Burley L, Sheats J, Murphy FG, et al. Dietary intake to reduce cancer risk among African American women in public housing: do sociodemographic factors make a difference? Ethn Dis. 2006;16:963–70. 17061754

[23] Beydoun MA, Wang Y (2008). Do nutrition knowledge and beliefs modify the association of socio-economic factors and diet quality among US adults?. Prev Med (Baltim).

[24] US Department of Health & Human Services. Healthy Food Financing Initiative Projects. HHS-2014-ACF-OCS-EE-0819. Washington (DC): Office of Community Services; Community Economic Development. [http://www.acf.hhs.gov/grants/open/foa/files/HHS-2014-ACF-OCS-EE-0819_1.pdf]. Accessed 21 Feb 2016.

[25] Drewnowski A, Darmon N (2005). Food choices and diet costs: an economic analysis. J Nutr.

[26] Edmonds J, Baranowski T, Baranowski J, Cullen KW, Myres D (2001). Ecological and socioeconomic correlates of fruit, juice, and vegetable consumption among African-American boys. Prev Med (Baltim).

[27] U.S. Department of Health and Human Services. Healthy People 2010: Understanding and Improving Health. 2nd ed. Washington, DC: U.S. Government Printing Office; 2000. http://www.healthypeople.gov/2010/document/pdf/uih/2010uih.pdf. Accessed10 Apr 2016.

[28] Treuhaft S, Karpyn A. The Grocery Gap: Who Has Access to Healthy Food and Why It Matters. Oakl CA PolicyLink Food Trust. 2010;29:473–80.

[29] Powell LM, Slater S, Mirtcheva D, Bao Y, Chaloupka FJ (2007). Food store availability and neighborhood characteristics in the United States. Prev Med (Baltim).

[30] Community Health Councils Inc. Does Race Define What’s in the Shopping Cart? Community Health & Education Policy Brief;2008. http://www.chc-inc.org/downloads/Shopping%20Cart%20Brief.pdf. Accessed 10 Apr 2016.

[31] Bodor JN, Rose D, Farley TA, Swalm C, Scott SK (2008). Neighbourhood fruit and vegetable availability and consumption: the role of small food stores in an urban environment. Public Health Nutr.

[32] Azuma A. Food access in central and south Los Angeles: Mapping injustice, agenda for action. Urban and Environmental Policy Institute; 2007. Retrieved from http://scholar.oxy.edu/uep_faculty/346. Accessed 10 Apr 2016.

[33] Franco M, Diez Roux AV, Glass TA, Caballero B, Brancati FL (2008). Neighborhood characteristics and availability of healthy foods in Baltimore. Am J Prev Med.

[34] Glanz K, Sallis JF, Saelens BE, Frank LD (2007). Nutrition Environment Measures Survey in stores (NEMS-S): development and evaluation. Am J Prev Med.

[35] Horowitz CR, Colson KA, Hebert PL, Lancaster K (2004). Barriers to buying healthy foods for people with diabetes: evidence of environmental disparities. Am J Public Health.

[36] Hosler AS, Varadarajulu D, Ronsani AE, Fredrick BL, Fisher BD (2006). Low-fat milk and high-fiber bread availability in food stores in urban and rural communities. J Public Health Manag Pract.

[37] Jetter KM, Cassady DL (2006). The availability and cost of healthier food alternatives. Am J Prev Med.

[38] Lexington Community Food Assesssment. [http://cfaky.org/test/wp-content/uploads/2015/03/CommunityFoodAssessmentReport04-07.pdf]. Accessed 21 Feb 2016.

[39] Gordon C, Ghai N, Purciel M, Talwalker A, Goodman A. Eating Well in Harlem: How Available Is Healthy Food? New York, NY: New York City Department of Health and Mental Hygiene; 2007.

[40] Alliance CF. Bridging the divide: Growing self-sufficiency in our food supply. Community Food Assessment: A Regional Approach for Food Systems in Louisville, Kentucky, Community Farm Alliance, Louisville, KY. 2007;52.

[41] Conroy D, McDavis-Conway S. Healthy Food, Healthy Communities: An Assessment and Scorecard of Community Food Security In the District of Columbia. D.C. Hunger Solutions, Washington, D.C. 2006;1–47.

[42] Cassady D, Jetter KM, Culp J (2007). Is price a barrier to eating more fruits and vegetables for low-income families?. J Am Diet Assoc.

[43] Drewnowski A (2004). Obesity and the food environment. Am J Prev Med.

[44] Glanz K, Hoelscher D (2004). Increasing fruit and vegetable intake by changing environments, policy and pricing: restaurant-based research, strategies, and recommendations. Prev Med (Baltim).

[45] Caldwell EM, Miller Kobayashi M, DuBow WM, Wytinck SM (2009). Perceived access to fruits and vegetables associated with increased consumption. Public Health Nutr.

[46] Haynes-Maslow L, Parsons SE, Wheeler SB, Leone LA (2013). A qualitative study of perceived barriers to fruit and vegetable consumption among low-income populations, North Carolina, 2011. Prev Chronic Dis.

[47] Lucan SC, Barg FK, Long JA (2010). Promoters and barriers to fruit, vegetable, and fast-food consumption among Urban, lowincome African Americans-a qualitative approach. Am J Public Health.

[48] Appleton KM, McGill R, Neville C, Woodside JV (2010). Barriers to increasing fruit and vegetable intakes in the older population of Northern Ireland: low levels of liking and low awareness of current recommendations. Public Health Nutr.

[49] Zepeda L, Reznickova A, Lohr L (2014). Overcoming challenges to effectiveness of mobile markets in US food deserts. Appetite.

[50] Dye CJ, Cason KL (2005). Perceptions of older, low-income women about increasing intake of fruits and vegetables. J Nutr Elder.

[51] Treiman K, Freimuth V, Damron D, Lasswell A, Anliker J, Havas S, et al. Attitudes and Behaviors Related to Fruits and Vegetables among Low-income Women in the WIC Program. J Nutr Educ. 1996;28:149–56.

[52] Anderson AS, Cox DN, McKellar S, Reynolds J, Lean ME, Mela DJ (1998). Take Five, a nutrition education intervention to increase fruit and vegetable intakes: impact on attitudes towards dietary change. Br J Nutr.

[53] Eikenberry N, Smith C (2004). Healthful eating: perceptions, motivations, barriers, and promoters in low-income Minnesota communities. J Am Diet Assoc.

[54] Gibson EL, Wardle J, Watts CJ (1998). Fruit and vegetable consumption, nutritional knowledge and beliefs in mothers and children. Appetite.

[55] Steptoe A, Perkins-Porras L, Rink E, Hilton S, Cappuccio FP (2004). Psychological and social predictors of changes in fruit and vegetable consumption over 12 months following behavioral and nutrition education counseling. Health Psychol.

[56] Henry H, Reimer K, Smith C, Reicks M (2006). Associations of decisional balance, processes of change, and self-efficacy with stages of change for increased fruit and vegetable intake among low-income, African-American mothers. J Am Diet Assoc.

[57] Promoting Healthy Eating and Physical Activity for a Healthier Nation. [http://www.cdc.gov/healthyyouth/publications/pdf/pp-ch7.pdf]. Accessed 21 Feb 2016.

[58] Booth SL, Sallis JF, Ritenbaugh C, Hill JO, Birch LL, Frank LD, et al. Environmental and societal factors affect food choice and physical activity: rationale, influences, and leverage points. Nutr Rev. 2001;59(3 Pt 2):S21–39. discussion S57–65. 10.1111/j.1753-4887.2001.tb06983.x 11330630

[59] Seymour JD, Yaroch AL, Serdula M, Blanck HM, Khan LK (2004). Impact of nutrition environmental interventions on point-of-purchase behavior in adults: a review. Prev Med (Baltim).

[60] Calancie L, Leeman J, Jilcott Pitts SB, Khan LK, Fleischhacker S, Evenson KR, et al. Nutrition-related policy and environmental strategies to prevent obesity in rural communities: a systematic review of the literature, 2002–2013. Prev Chronic Dis. 2015;12:E57. 10.5888/pcd12.140540 PMC4416478 25927605

[61] Larsen K, Gilliland J (2009). A farmers’ market in a food desert: Evaluating impacts on the price and availability of healthy food. Health Place.

[62] Payne GH, Wethington H, Olsho L, Jernigan J, Farris R, Walker DK (2013). Implementing a farmers’ market incentive program: perspectives on the New York City Health Bucks Program. Prev Chronic Dis.

[63] The National Mobile Market. [http://www.nationalmobilemarket.org]. Accessed 21 Feb 2016.

[64] Huang TT, Drewnosksi A, Kumanyika S, Glass TA (2009). A systems-oriented multilevel framework for addressing obesity in the 21st century. Prev Chronic Dis.

[65] Fitzgerald N, Spaccarotella K (2009). Barriers to a Healthy Lifestyle : From Individuals to Public Policy — An Ecological Perspective. J Ext.

[66] Dibsdall LA, Lambert N, Bobbin RF, Frewer LJ (2003). Low-income consumers’ attitudes and behaviour towards access, availability and motivation to eat fruit and vegetables. Public Health Nutr.

[67] Krueger R (1994). Focus Groups: A Practial Guide for Applied Research.

[68] Sallis JF, Owen N, Glanz K, Rimer B, Lewis F (2002). Ecological Models of Health behavior. Health Behavior and Health Education: theory, research, and practice.

[69] Stokols D (1996). Translating social ecological theory into guidelines for community health promotion. Am J Health Promot.

[70] McLeroy KR, Bibeau D, Steckler A, Glanz K (1988). An ecological perspective on health promotion programs. Health Educ Q.

[71] Baranowski T, Perry CL PG, Glanz K, Lewis FM RB (2002). How Individuals, environments, and health behaviors interact: social cognitive theory. Health Behavior and Health Education: Theory, Research and Practice.

[72] Bandura A. Social Foundations of Thought and Action: A Social Cognitive Theory. New Jersey: Prentice-Hall; Englewood Cliffs; 1986.

[73] Bandura A (1989). Human agency in social cognitive theory. Am Psychol.

[74] Gorham G, Dulin-Keita A, Risica PM, Mello J, Papandonatos G, Nunn A, et al. Effectiveness of Fresh to You, a Discount Fresh Fruit and Vegetable Market in Low-Income Neighborhoods, on Children’s Fruit and Vegetable Consumption, Rhode Island, 2010–2011. Prev Chronic Dis. 2015;12:E176. 10.5888/pcd12.140583 PMC4611858 26469949

[75] Yaroch AL, Tooze J, Thompson FE, Blanck HM, Thompson OM, Colón-Ramos U, et al. Evaluation of three short dietary instruments to assess fruit and vegetable intake: the National Cancer Institute’s food attitudes and behaviors survey. J Acad Nutr Diet. 2012;112:1570–7. 10.1016/j.jand.2012.06.002 PMC3775662 23017567

[76] Subar AF, Thompson FE, Kipnis V, Midthune D, Hurwitz P, McNutt S, et al. Comparative validation of the Block, Willett, and National Cancer Institute food frequency questionnaires : the Eating at America’s Table Study. Am J Epidemiol. 2001;154:1089–99. 10.1093/aje/154.12.1089 11744511

[77] Quan T, Salomon J, Nitzke S, Reicks M (2000). Behaviors of low-income mothers related to fruit and vegetable consumption. J Am Diet Assoc.

[78] Satia JA, Kristal AR, Patterson RE, Neuhouser ML, Trudeau E (2002). Psychosocial factors and dietary habits associated with vegetable consumption. Nutrition.

[79] Stables GJ, Subar AF, Patterson BH, Dodd K, Heimendinger J, Van Duyn MAS, et al. Changes in vegetable and fruit consumption and awareness among US adults: results of the 1991 and 1997 5 A Day for Better Health Program surveys. J Am Diet Assoc. 2002;102:809–17. 10.1016/s0002-8223(02)90181-1 12067046

[80] Thompson OM, Yaroch AL, Moser RP, Finney Rutten LJ, Petrelli JM, Smith-Warner SA, et al. Knowledge of and Adherence to Fruit and Vegetable Recommendations and Intakes: Results of the 2003 Health Information National Trends Survey. J Health Commun. 2011;16:328–40. 10.1080/10810730.2010.532293 21161813

[81] Shaikh AR, Yaroch AL, Nebeling L, Yeh M-C, Resnicow K (2008). Psychosocial predictors of fruit and vegetable consumption in adults a review of the literature. Am J Prev Med.

[82] Gattshall ML, Shoup JA, Marshall JA, Crane LA, Estabrooks PA (2008). Validation of a survey instrument to assess home environments for physical activity and healthy eating in overweight children. Int J Behav Nutr Phys Act.

[83] Steptoe A, Wijetunge S, Doherty S, Wardle J (1996). Stages of change for dietary fat reduction: associations with food intake, decisional balance and motives for food choice. Health Educ J.

[84] Wolf RL, Lepore SJ, Vandergrift JL, Wetmore-Arkader L, McGinty E, Pietrzak G, et al. Knowledge, barriers, and stage of change as correlates of fruit and vegetable consumption among urban and mostly immigrant black men. J Am Diet Assoc. 2008;108:1315–22. 10.1016/j.jada.2008.05.011 PMC3422563 18656571

[85] Harnack L, Block G, Subar A, Lane S, Brand R (1997). Association of cancer prevention-related nutrition knowledge, beliefs, and attitudes to cancer prevention dietary behavior. J Am Diet Assoc.

[86] Havas S, Treiman K, Langenberg P, Ballesteros M, Anliker J, Damron D, et al. Factors associated with fruit and vegetable consumption among women participating in WIC. J Am Diet Assoc. 1998;98:1141–8. 10.1016/S0002-8223(98)00264-8 9787720

[87] Townsend MS, Kaiser LL (2005). Development of a tool to assess psychosocial indicators of fruit and vegetable intake for 2 federal programs. J Nutr Educ Behav.

[88] Townsend MS, Kaiser LL (2007). Brief psychosocial fruit and vegetable tool is sensitive for the US Department of Agriculture’s Nutrition Education Programs. J Am Diet Assoc.

[89] Campbell MK, Reynolds KD, Havas S, Curry S, Bishop D, Nicklas T, et al. Stages of change for increasing fruit and vegetable consumption among adults and young adults participating in the national 5-a-Day for Better Health community studies. Health Educ Behav. 1999;26:513–34. 10.1177/109019819902600409 10435235

[90] Block G, Wakimoto P, Metz D, Fujii ML, Feldman N, Mandel R, et al. A randomized trial of the Little by Little CD-ROM: demonstrated effectiveness in increasing fruit and vegetable intake in a low-income population. Prev Chronic Dis. 2004;1:A08. PMC1253473 15670429

[91] Prochaska JO, DiClemente CC (1984). Self change processes, self efficacy and decisional balance across five stages of smoking cessation. Prog Clin Biol Res.

[92] Rhee KE, De Lago CW, Arscott-Mills T, Mehta SD, Davis RK (2005). Factors associated with parental readiness to make changes for overweight children. Pediatrics.

[93] Langenberg P, Ballesteros M, Feldman R, Damron D, Anliker J, Havas S (2000). Psychosocial factors and intervention-associated changes in those factors as correlates of change in fruit and vegetable consumption in the Maryland WIC 5 A Day Promotion Program. Ann Behav Med.

[94] Freedman DA, Choi SK, Hurley T, Anadu E, Hébert JR (2013). A farmers’ market at a federally qualified health center improves fruit and vegetable intake among low-income diabetics. Prev Med (Baltim).

[95] Abusabha R, Namjoshi D, Klein A (2011). Increasing access and affordability of produce improves perceived consumption of vegetables in low-income seniors. J Am Diet Assoc.

[96] Evans AE, Jennings R, Smiley AW, Medina JL, Sharma SV, Rutledge R, et al. Introduction of farm stands in low-income communities increases fruit and vegetable among community residents. Health Place. 2012;18:1137–43. 10.1016/j.healthplace.2012.04.007 22608130

[97] Jilcott Pitts SB, Wu Q, McGuirt JT, Crawford TW, Keyserling TC, Ammerman AS (2013). Associations between access to farmers’ markets and supermarkets, shopping patterns, fruit and vegetable consumption and health indicators among women of reproductive age in eastern North Carolina, U.S.A. Public Health Nutr.

[98] Herman DR, Harrison GG, Afifi AA, Jenks E (2008). Effect of a targeted subsidy on intake of fruits and vegetables among low-income women in the Special Supplemental Nutrition Program for Women, Infants, and Children. Am J Public Health.

[99] Anderson JV, Bybee DI, Brown RM, McLean DF, Garcia EM, Breer ML, et al. 5 a day fruit and vegetable intervention improves consumption in a low income population. J Am Diet Assoc. 2001;101:195–202. 10.1016/S0002-8223(01)00052-9 11271692

[100] Sifferlin A. Can “pop-up” grocery stores solve the problem of food deserts? Time 2012 (July 24)[http://healthland.time.com/2012/07/24/can-pop-up-grocery-stores-solve-the-problem-of-food-deserts]

[101] Widener MJ, Metcalf SS, Bar-Yam Y (2013). Agent-based modeling of policies to improve urban food access for low-income populations. Appl Geogr.

[102] Widener MJ, Metcalf SS, Bar-Yam Y (2012). Developing a mobile produce distribution system for low-income urban residents in food deserts. J Urban Health.

[103] Tester J, Yen I, Laraia B (2012). Using Mobile Fruit Vendors to Increase Access to Fresh Fruit and Vegetables for Schoolchildren. Prev Chronic Dis.

[104] Population Health Implications of the Affordable Care Act: Workshop Summary. Roundtable on Population Health Improvement;Board on Population Health and Public Health Practice; Institute of Medicine. Washington (DC): National Academies Press (US); 2014. http://www.nationalacademies.org/hmd/Reports/2013/Population-Health-Implications-of-the-Affordable-Care-Act.aspx. Accessed 21 Feb 2016. 25590107

[105] Trust for America's Health. Partner With Nonprofit Hospitals to Maximize Community Benefit Programs’ Impact on Prevention; 2013. http://healthyamericans.org/assets/files/Partner%20With%20Nonprofit%20Hospitals04. Accessed 10 Apr 2016.

[106] Compilation of Patient Protection and Affordable Care Act. Public Law 111–148; 2010. http://housedocs.house.gov/energycommerce/ppacacon.pdf. Accessed 10 Apr 2016.

[107] Health Care Without Harm. Utilization of Community Benefits to Improve Healthy Food Access in Massachusetts. Reston, VA: Health CareWithout Harm; 2015. http://www.wholesomewave.org/wp-content/uploads/2015/02/Utilization-of-Community-Benefits-to-Improve-Healthy-Food-Access-in-Massachusetts_FINAL.pdf.

[108] Fair Food Network: Double Up Food Bucks a Five-Year Success Story. [http://www.fairfoodnetwork.org/sites/default/files/FFN_DoubleUpFoodBucks_5YearReport.pdf]. Accessed 21 Feb 2016.

