# Supplementary figures and images for: A multi-level intervention in subsidized housing sites to increase fruit and vegetable access and intake: Rationale, design and methods of the ‘Live Well, Viva Bien’ cluster randomized trial
Source: BMC Public Health. 2016 Jun 28;16:521. doi: 10.1186/s12889-016-3141-7 (PMC4924350; doi:10.1186/s12889-016-3141-7)

Additional file 1: Photos of the mobile market


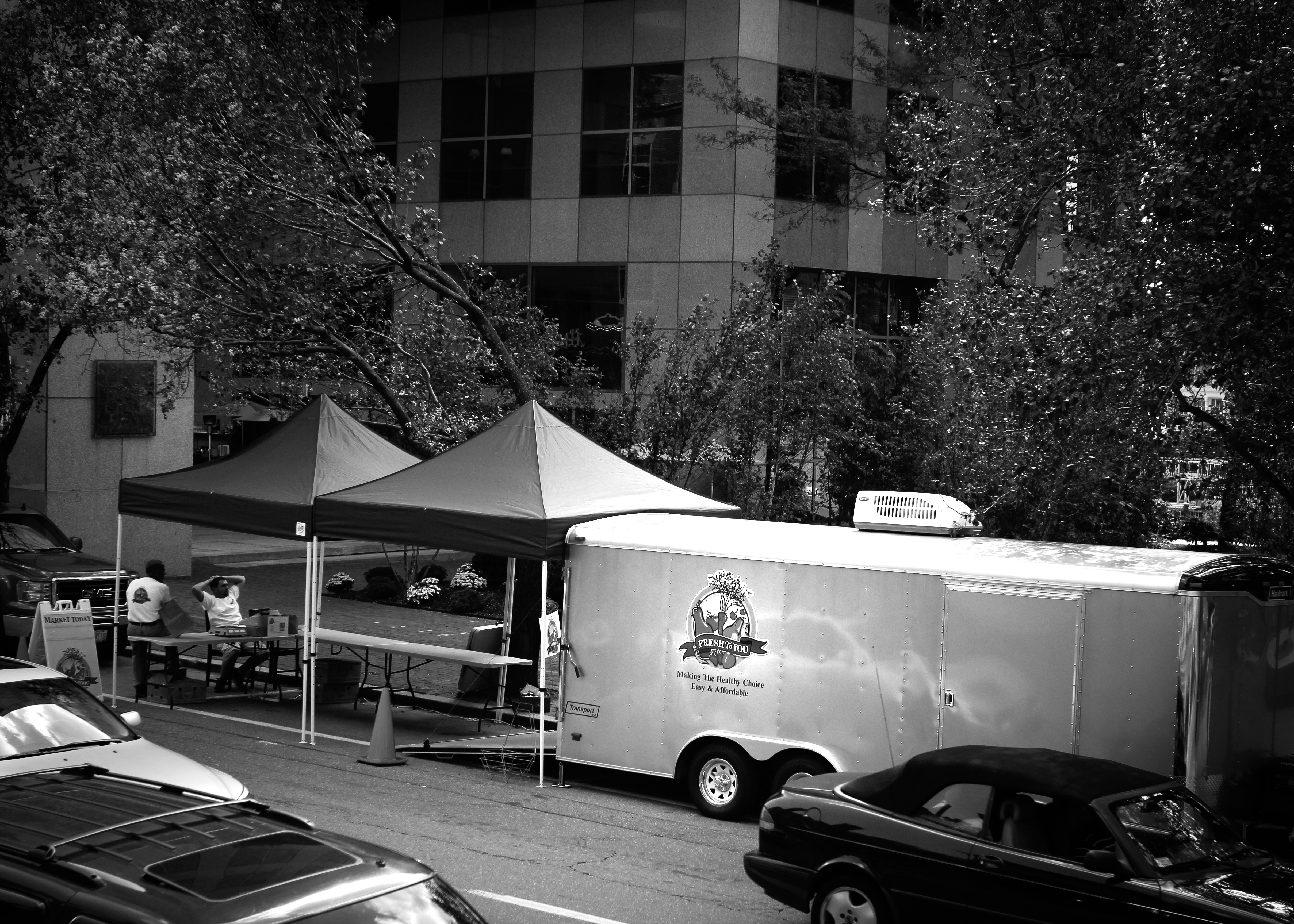

Supplement: Additional file 1: — Photos of the mobile market. (DOCX 7853 kb) [file 12889_2016_3141_MOESM1_ESM.docx]
